# Common subclinical hypothyroidism during Whipple’s disease

**DOI:** 10.1186/1471-2334-14-370

**Published:** 2014-07-04

**Authors:** Jean-Christophe Lagier, Florence Fenollar, Jacques Chiaroni, Christophe Picard, Christiane Oddoze, Laurent Abi-Rached, Didier Raoult

**Affiliations:** 1 Aix Marseille Université, URMITE, UM63, CNRS 7278, IRD 198, INSERM 1095, Faculté de Médecine, 27 Bd Jean Moulin, 13005 Marseille, France; 2 UMR 7268 (ADES), Aix-Marseille Université, CNRS, EFS, 51 Bd Pierre Dramard, 13916 Marseille, France; 3 APHM, CHU Timone, Laboratoire de Biochimie, 13005 Marseille, France; 4 Centre National de la Recherche Scientifique, Laboratoire d’Analyse, Topologie, Probabilités - Unité Mixte de Recherche 7353, Equipe ATIP, Aix-Marseille Université, 13331 Marseille, France

**Keywords:** *Tropheryma whipplei*, Whipple’s disease, Subclinical hypothyroidism, HLA

## Abstract

**Background:**

Classic Whipple’s disease is caused by *T. whipplei* and likely involves genetic predispositions, such as the *HLA* alleles DRB1*13 and DQB1*06, that are more frequently observed in patients. *T. whipplei* carriage occurs in 2-4% of the general population in France. Subclinical hypothyroidism, characterized by high levels of TSH and normal free tetra-iodothyronine (fT4) dosage, has been rarely associated with specific *HLA* factors.

**Methods:**

We retrospectively tested TSHus in 80 patients and 42 carriers. In cases of dysthyroidism, we tested the levels of free-T4 and anti-thyroid antibodies, and the HLA genotypes were also determined for seven to eight patients.

**Results:**

In this study, 72-74% of patients and carriers were male, and among the 80 patients, 14 (17%) individuals had a high level of TSH, whereas none of the carriers did (p < 0 · 01). In the 14 patients with no clinical manifestations, the T4 levels were normal, and no specific antibodies were present. Four patients treated with antibiotics, without thyroxine supplementation, showed normal levels of TSHus after one or two years. One patient displayed a second episode of subclinical hypothyroidism during a Whipple’s disease relapse five years later, but the subclinical hypothyroidism regressed after antibiotic treatment. HLA typing revealed nine alleles that appeared more frequently in patients than in the control cohort, but none of these differences reached significance due to the small size of the patient group.

**Conclusion:**

Regardless of the substratum, classic Whipple’s disease could lead to subclinical hypothyroidism. We recommend systematically testing the TSH levels in patients with Whipple’s disease.

## Background

Whipple’s disease, first described in 1907, is a chronic infectious disease caused by *Tropheryma whipplei*[1,2]. The positive diagnosis of classic Whipple’s disease is based on a positive histological assessment of a small bowel biopsy [1-3]. In addition to classic Whipple’s disease, *T. whipplei* also causes localized infections, such as endocarditis or encephalitis [1,4]. Although most individuals can eradicate the bacteria after a primo-infection (gastroenteritis or bacteremia), [5,6] others remain asymptomatic carriers [7], and an even smaller number of individuals develop chronic disease [8]. Genetic predispositions are strongly suspected in classic Whipple’s disease because human populations are commonly exposed to *T. whipplei*[9,10], yet individuals rarely develop classic Whipple’s disease. Consistently, classic Whipple’s disease mainly occurs in European middle-aged populations and has not been described in Sub-Saharan African populations, despite their high exposure to the bacterium [9]. Similarly, patients frequently relapse, and different *T. whipplei* strains can re-infect patients suffering from classic Whipple’s disease, suggesting a lifetime susceptibility to this bacterium [11-13]. Interestingly, a recent study highlighted that the *HLA* alleles DRB1*13 and DQB1*06 occurred significantly more frequently in patients with Whipple’s disease than in healthy individuals exposed to the bacteria [10].

Among the hypothyroidism substratum, a broad range of genetic defects has been reported, with different levels of clinical consequences ranging from severe congenital hypothyroidism [14-16] to unapparent manifestations in some cases of thyroid-stimulating hormone (TSH)-resistance [16]. Subclinical hypothyroidism is characterized by high TSH concentrations and normal serum thyroid hormones or serum free thyroid hormones. In the NHAES III study performed among US populations, the prevalence of subclinical hypothyroidism was 4 · 3%, associated with factors such as gender, age, body-mass index, and dietary iodine intake [17]. In addition, the prevalence of hyperthyroidism was higher in Europeans than in African Americans, suggesting that genetic factors also affect TSH secretion [17]. Among the causes of subclinical hypothyroidism, chronic lymphocytic thyroiditis (Hashimoto’s) represents 60 to 80% of the cases [17], but genetic factors, such as the *HLA* allele HLA-A*02, have also been associated with autoimmune thyroid disorders induced through interferon-alpha-therapy in patients with chronic hepatitis C, and an association of the HLA *A2-B46-DR9* haplotype with autoimmune thyroid dysfunction has also been described [10,18].

Some studies have reported the occurrence of hypothyroidism during Whipple’s disease [2,19,20]. Interestingly, a case of primary hypothyroidism with clinical manifestations was recently described, showing that the requirement for thyroxine supplementation dramatically and rapidly decreased after the initiation of antibiotic treatment; indeed, supplementation could be stopped after approximately 30 weeks, suggesting that *T. whipplei* directly infects the thyroid [19]. In addition to the apparent capacity of *T. whipplei* to infiltrate thyroid tissue [19], we hypothesized that the risk of developing subclinical hypothyroidism is also associated with host genetic factors.

Herein, we conducted a retrospective analysis of the TSH concentrations in 122 individuals with either classic Whipple’s disease (n = 80) or asymptomatic carriage of *T. whipplei* (n = 42). We also investigated the HLA types in patients suffering from hypothyroidism.

## Methods

### Patients

Since the first culture of *T. whipplei* in 2000, more than 27,000 *T. whipplei* PCR amplifications [21] have been performed at our research center for the diagnosis of more than 150 patients with classic Whipple’s disease [1]. Among these patients, serum was obtained from 80 patients with a clear diagnosis of classic Whipple’s disease. As a control, we used 42 patients with asymptomatic carriage of *T. whipplei* for which serum were available [7].

Definition of classic Whipple’s disease and asymptomatic carriage of *T. whipplei.*

A positive diagnosis of classic Whipple’s disease was obtained through the histological assessment (PAS staining and/or immunohistochemistry using antibodies specific for *T. whipplei*) of a small bowel biopsy specimen [2,3]. Asymptomatic carriers were defined as patients without clinical manifestations, showing positive *T. whipplei* PCR amplification from a stool sample [22].

### Laboratory findings

#### *Serum selection and preservation*

We tested retrospectively the TSHus levels in serum samples aliquoted and stored at -80°C because of the rarity of *T. whipplei* infections. To ensure that the storage time did not affect our analysis, we selected frozen samples from a wide range of times for the two groups studied: from 2003 to 2011 for asymptomatic carriers and from 2001 to 2012 for classic Whipple’s disease patients. The number of samples stored before or within the last five years was approximately equal in the two groups (Table 1).

**Table 1:** Baseline characteristics of patients and carriers (CWD = Classic Whipple’s disease)

	**CWD**	**Asymptomatic carriage of *T. whipplei***
**Number of cases**	80	42
**Male**	58 (72.5%)	31 (73.8%)
**Mean age, years (range)**	57.15 (28–84)	43.58 (18–80)
**Frozen serum (stored < 5 years)**	39 (48.1%)	19 (46.3%)
**Frozen serum (stored < 5 years among the higher TSH levels)**	6/14 (43%)	NA

#### *Serum tests*

The electrochemiluminescence immunoassay (ECLIA) for determination of TSH (thyroid-stimulating hormone), FT4 (free thyroxine hormone), anti-TPO (anti thyroid- specific peroxidase auto antibodies) and anti-Tg (antithyroid-specific thyroglobulin auto antibodies) levels was performed on a Cobas ® 6000 < e601 > analyzer (Roche Diagnostics, D-68298 Mannheim, France).

A sandwich immunoassay was used to detect the TSH levels, and a competition immunoassay was used to assess the levels of FT4, anti-TPO and anti-Tg. In these assays, the antigen-antibody complex binds to the solid phase via biotin and streptavidin interactions. The reaction mixture is subsequently aspirated into the measuring cell, where the microparticles are magnetically captured onto the surface of the electrode. The application of a voltage to the electrode induces chemiluminescent emission, measured using a photomultiplier.

### Normal serum level concentration

The serum level of TSH was normal when the concentration ranged from 0 · 27 to 4 · 20 mUI/L. The serum level of T4 was normal when the concentration ranged from 12 to 22 pmol/L. The serum level of anti-thyroid peroxidase antibodies and anti-thyroglobulin antibodies was considered normal for concentrations <115 UI/mL and <34 UI/mL, respectively.

### HLA analysis

Patients and blood donors from southeast France were genotyped for low resolution HLA-A, -B, -C, -DRB1 and –DQB1 loci using LABType SSO Typing Tests (ONE LAMBDA, INC, USA) according to the manufacturer’s instructions, and the results were analyzed using HLA Fusion v 1.2.1. software (ONE LAMBDA, INC, USA) for allele identification. The control cohort (n = 130) was obtained from southeast France (EFS Rhône Alpes, Histocompatibility Laboratory), and the data were obtained from the Allele frequency net database [23].

### Statistical analysis

**Figure 1:**
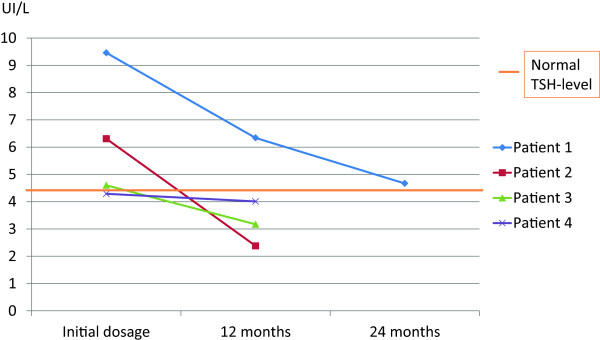
Time evolution of the TSH levels in four patients with CWD treated with antibiotics, without thyroxin supplementation.

Graph pad prism version 6.0 (La Jolla, USA) was used for the statistical analyses shown in Figure 1. Statistical significance was defined as *P* < 0 · 05. To exclude possible confounding effects, such as age or gender, a logistic regression model was used. The data analyses were conducted using SPSS v.20 (SPSS Inc., Chicago, IL, USA).

### Ethical statement

Informed consent was obtained from all the participating patients in the study. The study was conducted as part of normal care, without supplementary patient’s samples. The study was approved by the Ethics Committee of the Institut Fédératif de Recherche IFR48, Faculty of Medicine, Marseille France (agreement number 13–028).

## Results

### Patient’s characteristics

Among the 80 patients with classic Whipple’s disease and the 42 asymptomatic carriers, 58 (72 · 5%) and 31 (73 · 8%) individuals, respectively, were male, and the mean ages of the patient and carrier groups were 57 · 15 and 43 · 58 years, respectively.

### Laboratory results

**Figure 2:**
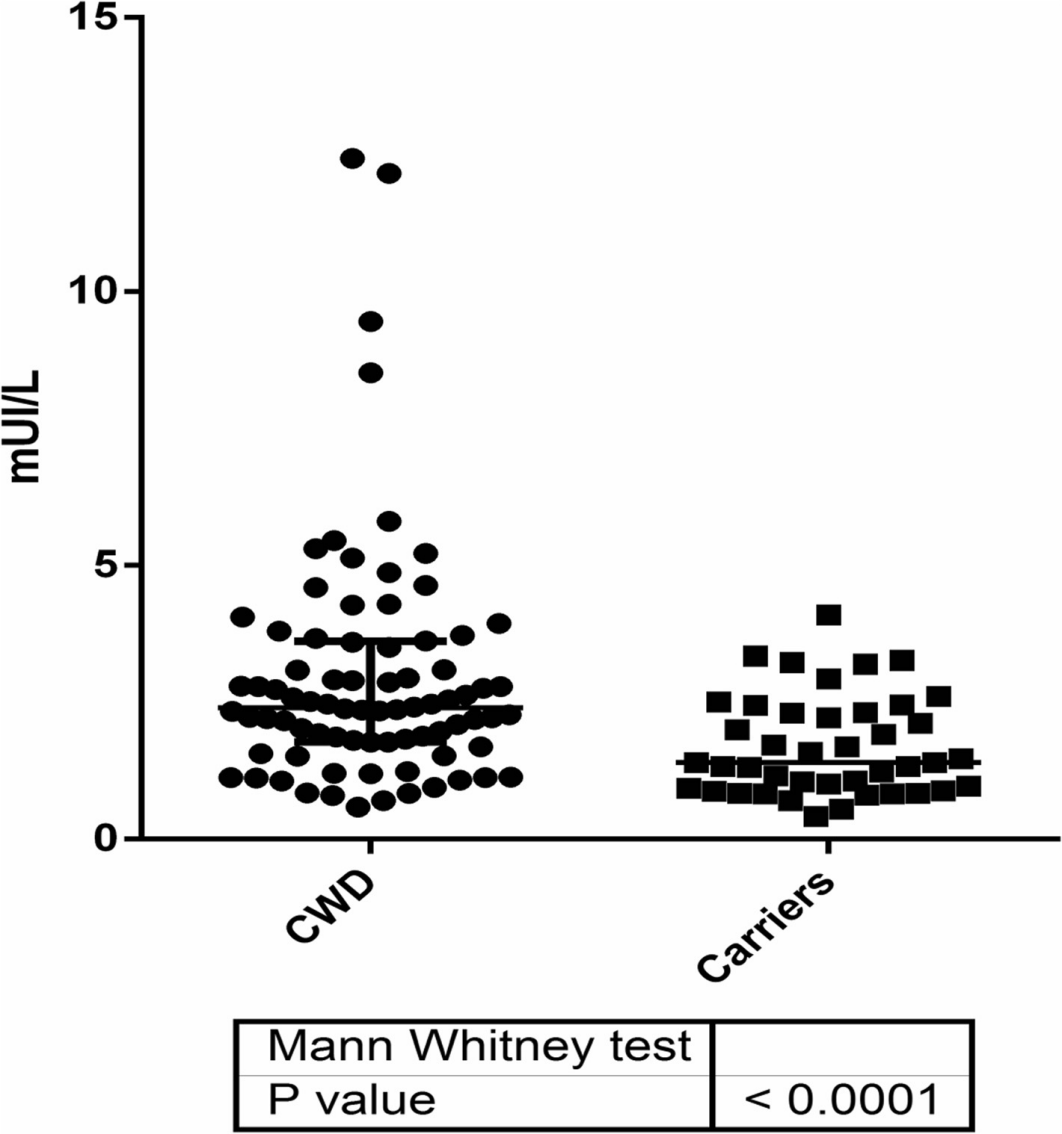
**TSH levels in the CWD and asymptomatic carrier groups.** The difference between the two groups is significant (*P* < 0.001).

Among the 80 patients with classic Whipple’s disease, 14 individuals (17.5%) showed high levels of TSH compared with the normal range (range: 4 · 28 to 12 · 43 mUI/L, mean: 6 · 58 mUI/L) but normal free T4 concentrations. Among these, 10 were male (71%) and the mean age was 58.28 years. None of these 14 individuals had another cause of increased TSH or other comorbidity than Whipple’s disease. Conversely, none of the 42 asymptomatic carriers showed an increase in the TSH concentration, resulting in a significant difference between the two groups (Figure 2). Using a multiple logistic regression analysis, we showed that TSH is significantly higher in classic Whipple’s disease, independent of the age of the patients (*P* < 0 · 01) (Table 2).

**Table 2:** Factors associated with CWD based on multiple logistic regression analyses

	**OR (95% CI)**	**p-value**
**TSH**	1.846 (1.186-2.875)	0.007
**Age**	1.058 (1.025-1.093)	0.001

Of the 14 patients with both classic Whipple’s disease and discrepancies in the TSH serum levels, 13 patients lacked positive anti-thyroglobulin and anti-thyroperoxidase antibodies (the 14th patient could not be tested because of an insufficient amount of serum) excluding a thyroiditis.

### Evolution under antibiotic treatment

We retrospectively tested four patients for the evolution of the TSH level after the initiation of antibiotic treatment for classic Whipple’s disease. Three of these four patients were treated with both doxycycline (200 mg/day) and hydroxychloroquine (600 mg/day), and the fourth patient was treated with trimethoprim-sulfamethoxazole (320 mg/1600 mg/day); none of these patients was treated with thyroxine supplementation. We observed a dramatic decrease in the TSH levels, particularly in the patient showing the highest TSH levels at the time of diagnosis (Figure 1).Interestingly, one of the patients experienced a relapse of Whipple’s disease at five years after cessation of the antibiotic treatment (Figure 1, Patient 1), and at the time of relapse, this patient also developed subclinical hypothyroidism (TSH = 7 · 15 UI/L, normal T3 and T4 serum levels). Three months after resuming the antibiotic treatment (doxycycline and hydroxychloroquine), the TSH levels were normal, although the patient had not been treated with thyroxin supplementation.

### HLA analysis

Among the 14 patients with classic Whipple’s disease and subclinical hypothyroidism, seven individuals were typed for *HLA class I*, and eight patients were typed for *HLA class II* (Table 3). A comparison of the HLA frequencies in these patients with those of a control cohort from southeast France [23] revealed that nine alleles were overrepresented in patients (odds ratio >3), including HLA-A*02, which was observed in 5 of the 7 patients (71%), but only in 55 of the 130 (42%) donors in the control group. Despite these large odds ratios, none of these comparisons reached significance due to the small size of the patient group.

**Table 3:** HLA-A, -B, -C, -DRB1 and -DQB1 types in patients with hypothyroidism

**HLA type**		**Number of individuals**				**Cohort vs. control**	
**Locus**	**Allele**	**Hypothyroidism**		**Control**		**Odds Ratio**	***P***^**1**^
HLA-A	01	0	0%	36	28%	N.A	0.19
	02	5	71%	55	42%	3.41	> 0.2
	03	2	29%	32	25%	1.23	
	24	2	29%	26	20%	1.60	
	26	1	14%	11	8%	1.80	
	29	1	14%	14	11%	1.38	
	32	1	14%	18	14%	1.04	
	68	2	29%	14	11%	3.31	0.19
	Total	7		130			
HLA-B	07	1	14%	19	15%	0.97	
	14	2	29%	12	9%	3.93	0.15
	15	2	29%	20	15%	2.20	
	27	1	14%	8	6%	2.54	
	35	1	14%	20	15%	0.92	
	38	1	14%	5	4%	4.17	> 0.2
	39	1	14%	6	5%	3.44	> 0.3
	41	1	14%	4	3%	5.25	> 0.2
	44	2	29%	34	26%	1.13	
	51	1	14%	15	12%	1.28	
	55	1	14%	8	6%	2.54	
	Total	7		130			
HLA-C	03	3	43%	26	20%	3.00	0.16
	04	1	14%	22	17%	0.82	
	05	2	29%	21	16%	2.08	
	06	1	14%	26	20%	0.67	
	07	3	43%	66	51%	0.73	
	08	2	29%	13	10%	3,60	0,17
	12	1	14%	18	14%	1,04	
	15	1	14%	7	5%	2,93	
	Total	7		130			
HLA-DRB1	01	2	25%	27	21%	2,29	
	03	1	13%	24	19%	0,63	
	04	2	25%	36	28%	0,87	
	07	1	13%	19	15%	0,83	
	08	1	13%	9	7%	1,92	
	11	3	38%	36	28%	1,57	
	13	3	38%	38	29%	1,45	
	14	1	13%	13	10%	1,29	
	15	1	13%	24	19%	0,63	
	Total	8		130			
HLA-DQB1	02	2	25%	37	29%	0,84	
	03	4	50%	93	72%	0,40	> 0.2
	04	1	13%	5	4%	3,57	> 0.3
	05	4	50%	48	37%	1,71	
	06	3	38%	56	43%	0,79	
	Total	8		130			

Odds ratios ≥3 are highlighted in light gray, and odds ratios <0.5 are highlighted in dark gray.

1: Two-tailed Fisher Exact Test.

## Discussion

**Figure 3:**
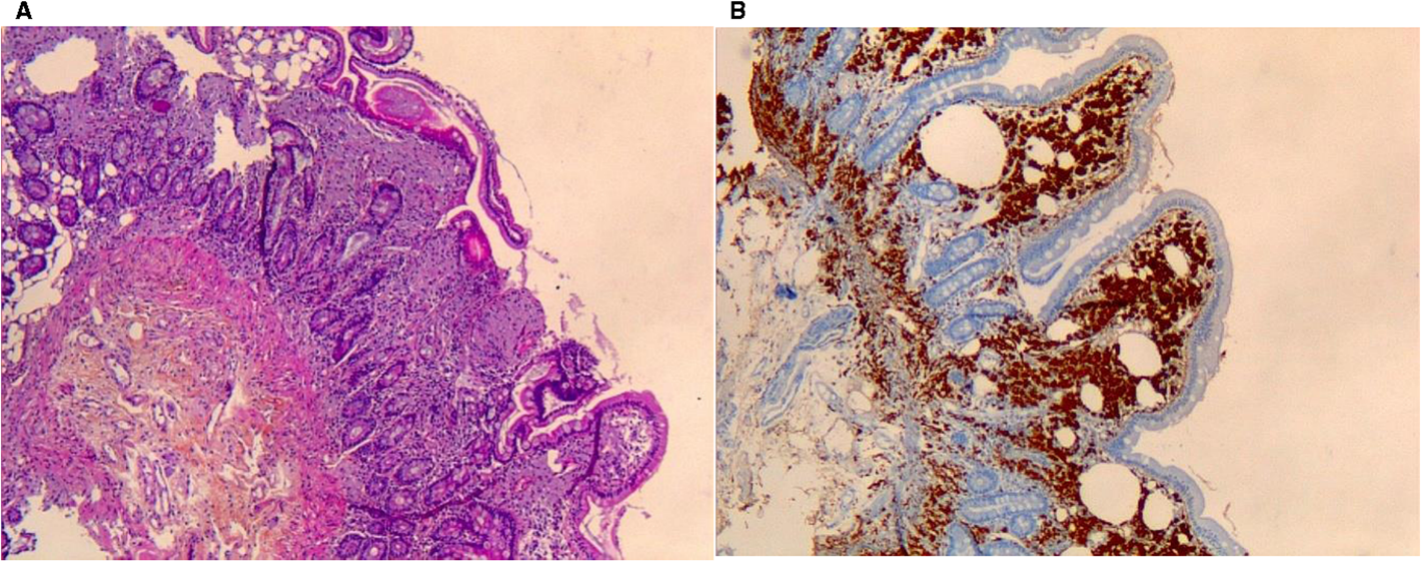
Histological assessment of a duodenal biopsy with positive periodic acid-Schiff staining (A) and positive immunohistochemical staining with polyclonal rabbit anti-T. whipplei antibody and Mayer’s haemalum counterstain (B).

Here, we described a significant association between patients suffering from classic Whipple’s disease and subclinical hypothyroidism. These results are particularly reliable because we used stringent diagnosis criteria to define classic Whipple’s disease, i.e., small bowel histological assessment (Figure 3) [3]. These findings were based on techniques routinely used in biochemical analyses according to the manufacturer's instructions. The influence of the serum preservation in our results was also minimized because we used frozen aliquots of serum stored at -80°C, representing the best preservation conditions, and the number of serum samples tested previously or within the last five years was comparable between the patient and carrier groups. Moreover, the proportion of recently sampled or previously stored serum was comparable among the 14 discrepant serum samples (Table 1). The two groups were also comparable in terms of gender proportion with a majority of male as previously known in Whipple’s disease [1], but older individuals were present in the patient group compared with the carrier group (Tables 1 and 2). Nevertheless, these results are significant because the multiple logistic regression analysis revealed that the association between higher TSH and classic Whipple’s disease is independent of the age of patients (Table 2). Finally, we observed a proportion of patients with subliclinical hypothyroidism (17%) significantly highest compared with general population (from 3% to 4%) [17].

The characteristics of the symptoms associated with subclinical hypothyroidism, which are neither sensitive nor specific [17] could explain the underdiagnosis of classic Whipple’s disease. Despite controversies regarding whether to treat with thyroxin supplementation in these cases, [17,24,25] the early detection of TSH disorders could reveal conditions associated with the risk of developing thyroid disease [17] and consequently an increasing risk of both cardiovascular diseases and heart failure. *T. whipplei* can spread to various tissues and fluids [1,2], leading to thyroid tissue infiltration, and this model is supported by the regression of biological discrepancies under adapted antibiotic treatment [19] and the elimination of other classic causes of subclinical hypothyroidism [17]. However, in previously described cases, thyroid cytology aspirations were PAS-negative, and the *T. whipplei* PCR amplification failed in two different samples [19]. A strong relationship between *T. whipplei* and subclinical hypothyroidism was demonstrated in a patient showing successive subclinical hypothyroidism during two different disease relapses, which was regressive in both cases after antibiotic initiation.

Hypothyroidism and classic Whipple’s disease have been recently associated with specific HLA types. Here, we observed an overrepresentation of nine *HLA* alleles (HLA-A*02, A*68, B*14, B*38, B*39, B*41, C*03, C*08, DQB1*04) (Table 3) in patients with classic Whipple’s disease and subclinical hypothyroidism, but these differences did not reach significance, likely reflecting the small number of patients investigated; thus, a larger cohort could confirm the results of Martinetti et al. [10] regarding HLA and Whipple’s disease and will be necessary to confirm these initial observations regarding an association with subclinical hypothyroidism .

## Conclusions

Despite the cause of subclinical hypothyroidism, in the present study, we demonstrated an increased risk in patients with classic Whipple’s disease that is not present in *T. whipplei* asymptomatic carriers. Consequently, we recommend both prospective and systematic testing of the TSH and T4 serum concentrations in patients with classic Whipple’s disease. Because of the subclinical nature and the reversibility under antibiotic treatment, we not recommend a thyroxin supplementation and to control after 1 month of antibiotics.

## Abbreviations

PAS: Periodic acid Schiff; FT4: Free thyroxine hormone; anti-TPO: Anti thyroid- specific peroxidase auto antibodies; anti-Tg: Antithyroid-specific thyroglobulin auto antibodies.

## Competing interests

JCL, FF, JC, CP, CO, LAR and DR declared no conflict of interests. The study was funded by IHU Mediteranee Infection that did not have roles in study design, data collection, analysis, interpretation and writing of the article.

## Authors’ contributions

DR designed the study, JCL, LAR and DR wrote the paper; JCL, FF, JC, CP and CO collected the data. JCL, FF, JC, CP, CO, LAR and DR interpreted the data, critically revised and approved the paper.

## Authors’ informations

MD, PhD JCL is an infectious disease specialist in IHU Méditerranée Infection Marseille France, MD, PhD FF is a clinical microbiologist in IHU Méditerranée Infection, Marseille France, MD, PhD Jacques Chiaroni is the director of Etablissement Français du sang, MD, PhD CP is a biologist, director of HLA-lab in Etablissement Français du sang, PharmD CO is a specialist of biochemistry in Assistance Publique Hopitaux de Marseille, PhD LAR is an immunologist, specialized in the understanding the principles underlying the evolution of vertebrate immune systems, Aix Marseille Université, MD PhD DR is a clinical microbiologist, director of IHU Méditerranée Infection Marseille France.

## Pre-publication history

The pre-publication history for this paper can be accessed here:

http://www.biomedcentral.com/1471-2334/14/370/prepub
